# Yellow Fever

**Published:** 1858-10

**Authors:** 


### YELLOW FEVER.

We continue to receive melancholy accounts of the ravages of Yellow Fever in our sister cities of the South. While we sympathize most deeply with those who are thus annually afflicted with this dread disease, we can but rejoice that there is a locality in a southern clime to which a large amount of the business naturally belonging to Yellow Fever cities (and which would now be in active operation but for the prevalence of this disease) might be transacted at any season of the year. Atlanta offers to the citizens of our coast cities not only a safe asylum from every malignant epidemic, but facilities of communication which it is thought should make it a point of attraction for wholesale merchants to bring their goods and distribute them, perhaps as profitably as from the sea board.

This plan, it seems to us, is the only one by which the process which is constantly going on, of concentration of every thing upon New York, can be arrested. At the very season when the merchants of the interior must and will buy their goods, the Southern coast cities are being scourged with Yellow Fever, and will not of course be resorted to, when their own population are flying from the pestilence.

The prospects of our city have never been so flattering, if we are to judge by the large number of substantial buildings which are going up in every direction and which are paying handsome rents, offering continued inducements to capitalists to invest their means in real estate here.

Not exceeded, by any locality upon the face of creation for health ; in a delightful climate (elevated about 1100 feet above the sea) equally free from the snows and ice of the North, and the excessive heat of the South, with Railroad communication in almost every direction, and having already attained a point in its existence beyond the apprehension of a retrograde movement, what can retard its progress?

---

☞ We are requested by the publishers, Messrs. S. S. & W. Wood, 389 Broadway, New York, to mention the prices at which several books recently noticed in our Journal may be obtained *by mail free of postage*:

"Nature and Art in the Cure of Disease, by Sir Jno. Forbes, M. D., &c." Price $1,00.

"Mind and Matter or Physiological Inquiries, by Sir Benjamin Brodie, &c." Price $1,00.

"Brown on Uremic Convulsions," (a valuable book to be noticed in the next number.) Price 75 cents.

